# TikTok and Attention-Deficit/Hyperactivity Disorder: A Cross-Sectional Study of Social Media Content Quality

**DOI:** 10.1177/07067437221082854

**Published:** 2022-02-23

**Authors:** Anthony Yeung, Enoch Ng, Elia Abi-Jaoude

**Affiliations:** 1 Department of Psychiatry, 12358 University of British Columbia, Vancouver, British Columbia, Canada; 2 Centre for Addiction and Mental Health (CAMH), Toronto, Ontario, Canada; 3 Department of Psychiatry, 12366 University of Toronto, Toronto, Ontario, Canada; 4 Department of Psychiatry, The Hospital for Sick Children, 12366 University of Toronto, Toronto, Ontario, Canada

**Keywords:** TikTok, social media, adhd, attention-deficit/hyperactivity disorder, misinformation, public health, e-mental health, diagnosis

## Abstract

**Objectives:**

Social media platforms are increasingly being used to disseminate mental health information online. User-generated content about attention-deficit/hyperactivity disorder (ADHD) is one of the most popular health topics on the video-sharing social media platform TikTok. We sought to investigate the quality of TikTok videos about ADHD.

**Method:**

The top 100 most popular videos about ADHD uploaded by TikTok video creators were classified as misleading, useful, or personal experience. Descriptive and quantitative characteristics of the videos were obtained. The Patient Education Materials Assessment Tool for Audiovisual Materials (PEMAT-A/V) and *Journal of American Medical Association* (JAMA) benchmark criteria were used to assess the overall quality, understandability, and actionability of the videos.

**Results:**

Of the 100 videos meeting inclusion criteria, 52% (*n* = 52) were classified as misleading, 27% (*n* = 27) as personal experience, and 21% (*n* = 21) as useful. Classification agreement between clinician ratings was 86% (kappa statistic of 0.7766). Videos on the platform were highly understandable by viewers but had low actionability. Non-healthcare providers uploaded the majority of misleading videos. Healthcare providers uploaded higher quality and more useful videos, compared to non-healthcare providers.

**Conclusions:**

Approximately half of the analyzed TikTok videos about ADHD were misleading. Clinicians should be aware of the widespread dissemination of health misinformation on social media platforms and its potential impact on clinical care.

## Abrégé

**Objectifs:**

Les plateformes des médias sociaux servent de plus en plus à diffuser de l’information sur la santé mentale en ligne. Le contenu créé par les utilisateurs au sujet du trouble de déficit de l’attention avec hyperactivité (TDAH) est un des thèmes de santé les plus populaires sur la plateforme de partage vidéo du média social TikTok. Nous avons cherché à investiguer la qualité des vidéos TikTok en matière de TDAH.

**Méthode:**

Les 100 vidéos les plus populaires sur le TDAH téléchargées par les créateurs de vidéo de TikTok ont été classées comme étant une expérience trompeuse, utile ou personnelle. Les caractéristiques descriptives et quantitatives des vidéos ont été obtenues. L’outil d’évaluation du matériel d’éducation des patients (PEMAT) pour le matériel audio-visuel (AV) et les critères de référence du Journal of American Medical Association (JAMA) ont servi à évaluer la qualité, la compréhensibilité et l’applicabilité générales des vidéos.

**Résultats:**

Sur les 100 vidéos qui satisfaisaient aux critères d’inclusion, 52 % (n = 52) étaient classées trompeuses, 27 % (n = 27) comme une expérience personnelle, et 21 % (n = 21) utiles. La concordance du classement entre les cotes des cliniciens était de 86 % (statistique kappa-de 0.7766). Les vidéos de la plateforme étaient très compréhensibles pour les spectateurs mais avaient une faible applicabilité. Les prestataires autres que de la santé ont téléchargé la majorité des vidéos trompeuses. Les prestataires de soins de santé ont téléchargé des vidéos de plus grande qualité et plus utiles, comparativement aux prestataires autres que de la santé.

**Conclusions:**

Presque la moitié des vidéos TikTok analysées sur le TDAH était trompeuse. Les cliniciens devraient être au courant de la diffusion et de la désinformation répandue en matière de santé sur les plateformes des médias sociaux et de son effet potentiel sur les soins cliniques. l.

## Introduction

Social media platforms are a popular means of sharing medical information online. TikTok in particular is a relatively new social media platform that has seen rapid adoption by adolescents and young adults, becoming the most downloaded social media application in 2020 with more than 1 billion monthly active users.^1,2^ In particular, the popularity of the platform appears to have contributed to increased awareness of attention-deficit/hyperactivity disorder (ADHD), with some individuals seeking a diagnosis after watching videos about ADHD on the platform.^3,4^ The hashtag “#adhd” is currently the seventh most popular health-related hashtag on the platform.^2^ Although social media can reduce mental health stigma and improve health literacy, there is also concern about misinformation and the potential for illness/health anxiety (“cyberchondria”) due to the volume of unmoderated, user-generated content online.^2,5,6^ For example, TikTok videos have been implicated in a recent rise of tic-like behaviours in adolescents, and it is thought that exposure to tic-related videos is responsible for this phenomenon.^7-9^

Additionally, social media platforms use proprietary algorithms that focus on increasing user engagement and may promote videos that do not necessarily reflect accurate health information. A recent systematic review found that the prevalence of health misinformation was high across almost all social media platforms.^6^ With TikTok being a relatively new social media platform, there is little research on the quality and accuracy of its content. As a result, there have been increasing calls for more research to understand the potential implications of TikTok on health information dissemination.^2^ Recent studies examining TikTok videos have found that misinformation was common for a variety of medical topics including those about acne, diabetes, and cosmetic surgery.^10-12^ To our knowledge, no study has examined the quality of mental health information disseminated on TikTok. Thus, our objective was to assess the quality of medical information in the most popular TikTok videos related to ADHD.

## Methods

**Figure 1.**
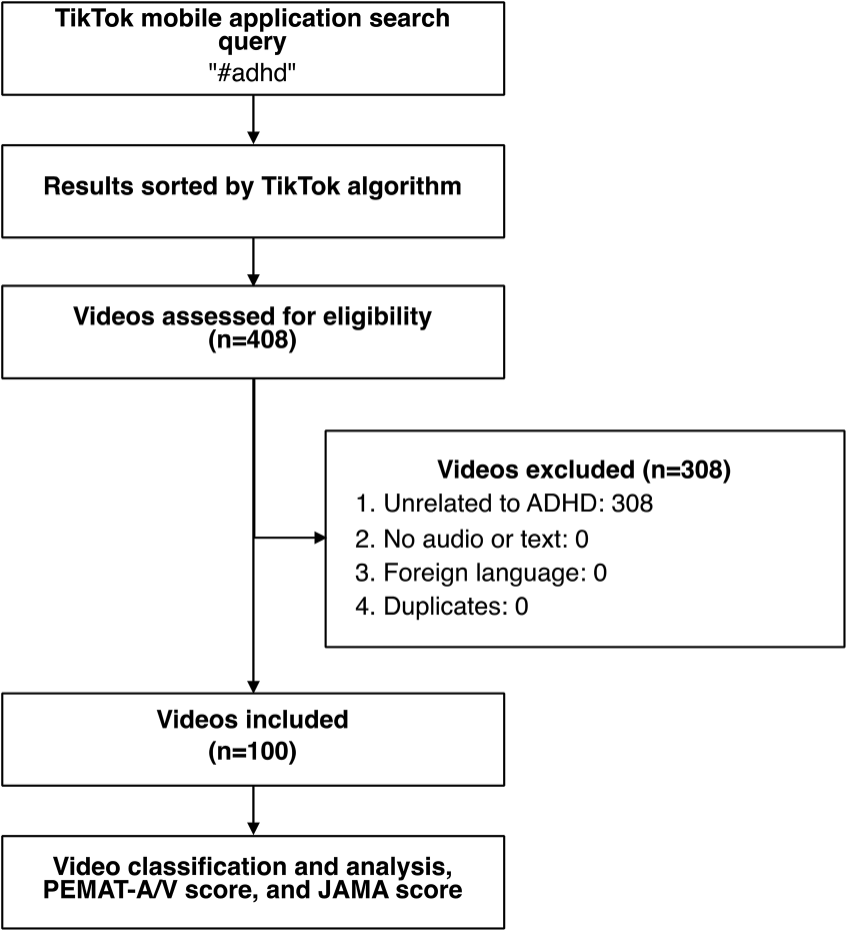
Flow chart of method of video inclusion.

The TikTok mobile phone application was queried with a search for the hashtag “#adhd” on July 18, 2021 (Figure 1). The proprietary search algorithm returns the most popular videos based on the number of views and likes per video. Our inclusion criteria were videos that specifically described or educated viewers about: ADHD symptoms/diagnosis, lived experience with ADHD, or ADHD management. Exclusion criteria were videos with no audio or text, non-English videos, videos unrelated to ADHD, or duplicate videos. Videos were sorted in descending order by view count, and the inclusion criteria were applied until 100 videos were identified. The sample size of 100 videos was chosen as a feasible and representative sample size to capture the most viewed videos on the platform. Previous studies have used similar sample sizes to analyze health-related videos on social media platforms such as TikTok and YouTube.^10,13-15^ Videos meeting the inclusion criteria were then included for extraction of video characteristics and further analysis.

One psychiatrist and one psychiatry resident (AY, EN) with clinical experience in the diagnosis and management of ADHD independently assessed the videos using a categorical classification often used to assess the content quality of social media videos.^16,17^ Videos were viewed, analyzed, and classified as: (1) useful; (2) personal experience; or (3) misleading. Videos were classified as useful if the video contained scientifically correct information about any aspect of ADHD: prevention, symptoms, diagnosis, treatments, or other information (e.g., etiology, psychopathology, epidemiology, prognosis). Videos were classified as personal experiences if it described a user's own personal or anecdotal experience of ADHD symptoms or treatment. Videos were classified as misleading if it contained information lacking scientific evidence (e.g., unsubstantiated claims about treating ADHD). If a personal experience video additionally contained any generalized misleading statements, it was classified as misleading rather than personal experience.

Where applicable, raters referenced the Canadian ADHD Resource Alliance (CADDRA) Canadian ADHD Practice Guidelines, Fourth Edition, and American Academy of Pediatrics ADHD Clinical Practice Guidelines.^18,19^ In cases where there was disagreement between the first two raters, a third psychiatrist rater (EAJ) independently rated the video to decide on the final rating.

All videos were also assessed using the Patient Education Materials Assessment Tool for Audiovisual Materials (PEMAT-A/V), a validated instrument to assess the understandability and actionability of audiovisual patient education videos.^20^ Finally, videos were also rated on the *Journal of American Medical Association* (JAMA) benchmark criteria,^21^ a four-point scale which assesses medical information quality based on: (1) authorship (all authors and contributors, their affiliations, and relevant credentials should be provided), (2) attribution (references and sources for all content should be listed clearly, and all relevant copyright information noted), (3) disclosure (any sponsorship, advertising, underwriting, commercial funding arrangements or support, or potential conflicts of interest disclosed), and (4) currency (dates that content was posted and updated are indicated).

Video metadata was extracted automatically using an open-source web scraper using TypeScript programming language (TikTok-scraper). Descriptive characteristics for each video included: user type (individual, organization, or healthcare provider [HCP]), number of views, number of likes, and number of shares. The kappa statistic was calculated to assess inter-rater reliability for the initial video classification between the two raters. Student's *t* test was used to compare video characteristics, PEMAT scores, and JAMA scores between HCPs and non-HCPs video uploaders. Pearson's chi-square test was used to compare frequencies of the video classifications between HCPs and non-HCPs. One-way analysis of variance was used to compare video characteristics, PEMAT scores, and JAMA scores between the three video classifications. A *P*-value of less than 0.05 was considered significant for all analyses. All statistical analyses were performed using Stata 17.0.

## Results

### Baseline Video Characteristics

Prior to applying the exclusion criteria, there were a total of 4.3 billion views on videos tagged with “#adhd” at the time of the query. The 100 videos meeting inclusion criteria had a total of 283,459,400 views; 89 videos were uploaded by non-HCPs, while 11 were uploaded by individuals identifying as HCPs (i.e., licensed therapists, nurses, psychologists, or physicians). None of the videos in this sample were uploaded by corporations, health organizations, for-profit entities, or non-profit entities. There was an average of 2.8 million views (range: 892,100–14,800,000) and 31,175 shares (range: 1,249–245,200) per video. The average video length was 36.7 s (range: 8.0–74). Across all videos, the average PEMAT-A/V understandability score was 94.3% (range: 66.7–100%), and the average PEMAT-A/V actionability score was 14.3% (range: 0–100%). The average JAMA benchmark score was 1.3 (range: 1–3).

### Videos Characteristics by Classification

The full video characteristics by the classification are described in Table 1. Of the 100 videos analyzed, 52% were classified as misleading, 27% as personal experience, and 21% as useful. Agreement between the raters on the classification was 86%, with a kappa statistic of 0.7766 (*P* < 0.001). Personal experience videos had the highest PEMAT-A/V understandability score, with an average score of 98.3% (*P* < 0.001), and were the most popular (based on the average number of likes, views, and shares).

**Table 1.** Video Characteristics by Classification.

	Misleading (*n* = 52)	Personal experience (*n* = 27)	Useful (*n* = 21)	*P* value
Mean Views (SD, range)	2,496,717 (1,533,720, 892,100–7,800,000)	3,870,485 (3,752,937, 903,100–14,800,000)	2,339,381 (1,707,814, 927,000–8,900,000)	<0.001
Mean Likes (SD, range)	549,165 (372,605, 294,700–2,100,000)	839,033 (779,499, 312,600–3,200,000)	566,742 (409,171, 298,000–2,100,000)	<0.001
Mean Shares (SD, range)	24,344 (25,918, 1,249–126,000)	46,200 (64,713, 5,293–245,200)	28,773 (32,391, 1,411–147,300)	<0.001
PEMAT-A/V Understandability (SD, range)	92.3% (9.3%, 66.7–100%)	98.3% (4.7%, 83.3–100%)	93.7% (9.1%, 71.4–100%)	<0.001
PEMAT-A/V Actionability (SD, range)	13.1% (30.8%, 0–100%)	6.2% (18%, 0–66.7%)	27.8% (42.3%, 0–100%)	<0.001
JAMA Score (SD, range)	1.2 (0.5, 1–3)	1.2 (0.4, 1–2.5)	1.7 (0.7, 1–3)	0.005

*Note.* HCP = Healthcare provider; PEMAT-A/V = Patient Education Materials Assessment Tool for Audiovisual Materials; JAMA = *Journal of American Medical Association*.

Table 2 provides illustrative examples of useful, personal experience, and misleading videos that were included for analysis. Of the 52 misleading videos, 37 videos (71%) misattributed transdiagnostic psychiatric symptoms as being specific only to ADHD, including anxiety, depression, anger, relationship conflicts, dissociation, and mood swings. None of the misleading videos recommended viewers to seek out a medical, psychiatric, or psychological assessment before attributing these symptoms to ADHD. Eight videos (15%) misrepresented the pathophysiology of ADHD, including oversimplifying the disorder as a purely dopamine-deficient state. Four videos (7%) provided incorrect information about the approach to diagnosing ADHD, such as including an audio quiz to determine whether an individual has ADHD. Two videos (4%) misrepresented the association between ADHD and developmental theories, such as stating that individuals with ADHD lack object permanence. Finally, one video (2%) suggested non-validated coping strategies for ADHD with no evidence base.

**Table 2.** Illustrative Examples of Misleading, Useful, and Personal Experience Videos.

**Examples of misleading videos**
Video describing “ADHD paralysis” as an ADHD symptom where the brain “physically won't let me do anything” and “sometimes nothing causes it.”
Video stating that ADHD is “equally common between girls and boys” and that ADHD symptoms “only intensify with onset of puberty.”
Video stating that individuals with ADHD lack “object permanence.”
Video stating that “anxiety shivers,” “random noise making,” and “being competitive” are symptoms of ADHD.
Video stating individuals with ADHD are “only either understimulated or overstimulated” and “lack dopamine.”
**Examples of useful videos**
Video describing that auditory processing disorder can be a comorbid disorder with ADHD, and that a medical and/or audiological assessment is required for this to be diagnosed.
Video explaining that individuals with ADHD may need extra time to find missing items, have anxiety about future tasks, and suggests that proper treatment can improve quality of life.
Video stating that individuals with ADHD may have sleep disturbances and comorbidities such as delayed sleep phase syndrome.
Video describing ADHD signs and symptoms such as daydreaming, sleep disturbances, and school difficulties, with a recommendation to see a mental health professional if the viewer is wondering about ADHD.
Video describing ADHD signs and symptoms such as needing others to repeat information, forgetfulness, or struggling to complete tasks.
**Examples of personal experience videos**
Video of an individual describing their day of experiencing ADHD symptoms, such as being distracted while doing tasks and losing items.
Video of an individual describing their own personal experience of inattentive ADHD symptoms including self-doubt, feeling overwhelmed, and thoughts of procrastinating.
Video of an individual describing their personal experience of being forgetful, easily distracted, and also experiencing stigma related to the diagnosis of ADHD
Video of an individual with ADHD describing their own personal experience of having difficulty completing individual projects, and preference for group projects.
Video of an individual with ADHD showing a recording of themselves being distracted while performing a task.

*Note.* ADHD: attention-deficit/hyperactivity disorder.

Of the 21 useful videos, 17 videos (81%) described signs and symptoms of ADHD specific to the disorder, or informed viewers that transdiagnostic symptoms may be due to other medical conditions or comorbid psychiatric disorders. Three videos (14%) provided information on treatments or strategies for coping with ADHD that have been validated. One video (5%) accurately described the prognosis and risk factors for ADHD. Of the 27 personal experience videos, all videos described individual experiences of living with ADHD symptoms and/or its impact on day-to-day life.

### Videos Characteristics by Healthcare Providers and Non-Healthcare Providers

The full descriptive characteristics of the videos by creator type (HCP vs. non-HCP) are described in Table 3. Non-HCPs had significantly more misleading (55.1% vs. 27.3%, *P* < 0.001) and personal experience videos (29.2% vs. 9.1%, *P* < 0.001) than HCPs. HCPs had significantly more useful videos than non-HCPs (63.6% vs. 15.7%, *P* < 0.001). Non-HCP videos were more popular, though this was not statistically significant.

**Table 3.** Video Characteristics by Creator Type.

	Non-HCP (*n* = 89)	HCP (*n* = 11)	All (*n* = 100)	*P* value
Mean Views (SD, range)	2,957,553 (2,540,059, 892,100–14,800,000)	1,839,745 (675,059, 937,200–3,200,000)	2,834,594 (2,429,941, 892,100–14,800,000)	0.151
Mean Likes (SD, range)	659,344 (554,075, 294,700–3,200,000)	402,773 (130,453, 300,200–774,300)	631,121 (530,204, 294,700–3,200,000)	0.131
Mean Shares (SD, range)	32,603 (43,914, 1,249–245,200)	19,619 (11,892, 4,487–42,200)	31,175 (41,775, 1,249–245,200)	0.333
PEMAT-A/V Understandability (SD, range)	93.9% (8.7%, 66.7–100%)	97.2% (6.3%, 83.3–100%)	94.3% (8.5%, 66.7–100%)	0.229
PEMAT-A/V Actionability (SD, range)	12.9% (30.9%, 0–100%)	25.8% (35.3%, 0–83.3%)	14.3% (31.5%, 0–100%)	0.204
JAMA Score (SD, range)	1.2 (0.4, 1–2.5)	2.2 (0.6, 1–3)	1.3 (0.6, 1–3)	<0.001
Misleading, *n* (%)	49 (55.1%)	3 (27.3%)	52 (52%)	<0.001
Personal Experience, *n* (%)	26 (29.2%)	1 (9.1%)	27 (27%)	<0.001
Useful, *n* (%)	14 (15.7%)	7 (63.6%)	21 (21%)	<0.001

*Note.* JAMA = Journal of American Medical Association.

## Discussion

In this analysis of popular TikTok videos about ADHD, there were over 2.8 million views per video and each video was shared on average 31,000 times. Approximately half of the videos analyzed (52%) were misleading, with non-HCPs uploading most of these videos (49 out of 52 videos). There was substantial agreement among raters on the classification of the videos as useful, misleading, or personal experience. Our study is the first to show that misleading videos about ADHD are being widely disseminated and viewed on TikTok.

These findings are in line with previous studies of TikTok videos that have found high rates of misinformation for medical conditions, including acne and diabetes.^10,11^ Our results are also similar to a study by Thapa et al. that analyzed YouTube videos about ADHD and found that 38% of analyzed videos were misleading and only 5% were useful.^22^ The study also found that first-person or personal experience videos received the highest engagement (i.e., likes and views), while misleading videos were the most common. Our study replicated these findings on TikTok: personal experience videos received the highest engagement (i.e., views, likes, and shares), while misleading videos were the most common. These consistent findings across different social media platforms suggest that viewers are most drawn to videos made by individuals with lived experience, and less so towards institutional or HCP-created videos. Increasingly, social media influencers and independent video creators have been found to have an outsized influence on the dissemination of health information online.^23-25^ Indeed, in our study, none of the top 100 videos were uploaded by corporations, health organizations, for-profit entities, or non-profit entities, and only 11% of videos were uploaded by HCPs. This limited presence of HCPs on TikTok has been observed in other studies as well.^10^ Overall, HCPs did score significantly higher on the JAMA benchmark criteria and made significantly more useful videos and less misleading videos. Although this suggests that healthcare providers do upload higher quality content on the platform, 27% of HCP videos (3 out of 11) were still rated as misleading.

We additionally found that all videos about ADHD were highly understandable, scoring over 90% on the PEMAT-A/V understandability score. The understandability score does not reflect accuracy, but merely that the information is presented in an understandable manner. Thus, individuals may be seeing videos about ADHD on the platform that are highly understandable and yet misleading. This was seen in our qualitative review of misleading videos, which often had an oversimplified or reductionist explanation of ADHD.

The findings from our study provide several important insights into the dissemination of medical information about ADHD on TikTok. Approximately half of all videos analyzed were misleading, and the misinformation they contain has the potential to contribute to health anxiety or lead to increased healthcare utilization.^26^ The proprietary TikTok algorithm has been found to have a propensity to show users similar videos over time, which may further propagate misleading videos.^27,28^ Additionally, viral trends on TikTok,^29^ “echo chambers” in social media,^30^ and romanticization of mental health symptoms^9,31^ may perpetuate the dissemination of misleading information on TikTok. Increased healthcare use may occur for individuals who seek clinical attention after viewing TikTok videos about ADHD, a phenomenon that has been widely documented by major news organizations.^3,4^ Since self-report of ADHD symptoms may be over-endorsed,^32^ there may be an increased risk for overdiagnosis or misdiagnosis in these individuals as well. In general, there has also been renewed interest over rising rates of ADHD diagnoses,^33-35^ and debate about the overdiagnosis of ADHD.^35^

TikTok has become particularly popular over the course of the COVID-19 pandemic. The pandemic context raises the question of whether individuals may be misattributing difficulties from pandemic public health measures (e.g., having difficulty with maintaining attention when viewing a screen in isolation for prolonged periods) to ADHD symptoms.^36,37^ One related example is the rapid increase in tic-like behaviours that have been observed over the course of the COVID-19 pandemic.^7,9^ Tic disorder specialists have seen a dramatic increase in referrals for tic-like behaviours in primarily adolescent females that are inconsistent with usual presentations of primary tic disorders. These presentations are thought to be most consistent with a diagnosis of functional neurological disorder, related to stressors over the course of the pandemic. The sociogenic propagation of tic-like behaviours across TikTok is a reflection of the impact of social media platforms on health behaviours.^7,9^

Limitations of our study include TikTok's proprietary search algorithm results, which exclude video ads and do not allow for systematic searching of all videos or deleted videos. Not all videos contained detailed information to identify whether an uploader was a health professional or not. Additionally, the current study only screened for the top 100 most popular videos. Less popular videos may have different content quality and characteristics not captured in this study. Also, the conceptualization of what is considered as a misleading, useful, or personal experience video was done from the perspective of raters who are HCPs. This perspective may not necessarily be in line with healthcare consumers or patients. For instance, while some videos may not be useful from the perspective of an HCP, others may find it useful for other reasons. Additionally, although our inter-rater agreement was high, videos classified as useful could potentially be classified as personal experience videos. Videos on the platform may also be recorded to be humorous or spontaneous, and not necessarily recorded with the intent to disseminate medical information. However, even videos made without intent to disseminate medical information may describe non-specific symptoms, overgeneralizations, and characterizations about ADHD that could be misleading to viewers. Finally, there have also been criticisms of the use of tools such as the JAMA benchmark criteria as a method to evaluate online videos.^38^ However, there are currently no other widely used or accepted methods of assessing medical information videos online.

In summary, this study is the first to show that misleading videos about ADHD are being widely disseminated on TikTok. Although the platform is the most popular social media application of 2020 and 2021, it is currently the least studied of the major social media platforms.^2^ It is thus important for clinicians to be aware of the dissemination of misleading videos on TikTok and the potential impact on clinical care. Future areas of research include a better understanding of the prevalence and nature of misinformation on TikTok for other mental health topics such as depression, anxiety, suicide, and self-harm. Our finding that HCPs generally upload higher quality and more useful videos deserves replication and further study to see whether HCP engagement on the platform could help correct misinformation regarding ADHD and other mental health disorders.

## Supplemental Material

sj-xls-1-cpa-10.1177_07067437221082854 - Supplemental material for TikTok and Attention-Deficit/Hyperactivity Disorder: A Cross-Sectional Study of Social Media Content Quality Click here for additional data file. Supplemental material, sj-xls-1-cpa-10.1177_07067437221082854 for TikTok and Attention-Deficit/Hyperactivity Disorder: A Cross-Sectional Study of Social Media Content Quality by Anthony Yeung, Enoch Ng and Elia Abi-Jaoude in The Canadian Journal of Psychiatry
